# Processing Distracting Non-face Emotional Images: No Evidence of an Age-Related Positivity Effect

**DOI:** 10.3389/fpsyg.2017.00591

**Published:** 2017-04-13

**Authors:** Mark Madill, Janice E. Murray

**Affiliations:** Department of Psychology, University of OtagoDunedin, New Zealand

**Keywords:** older adults, selective attention, emotion processing, positivity effect, affective images

## Abstract

Cognitive aging may be accompanied by increased prioritization of social and emotional goals that enhance positive experiences and emotional states. The socioemotional selectivity theory suggests this may be achieved by giving preference to positive information and avoiding or suppressing negative information. Although there is some evidence of a positivity bias in controlled attention tasks, it remains unclear whether a positivity bias extends to the processing of affective stimuli presented outside focused attention. In two experiments, we investigated age-related differences in the effects of to-be-ignored non-face affective images on target processing. In Experiment 1, 27 older (64–90 years) and 25 young adults (19–29 years) made speeded valence judgments about centrally presented positive or negative target images taken from the International Affective Picture System. To-be-ignored distractor images were presented above and below the target image and were either positive, negative, or neutral in valence. The distractors were considered task relevant because they shared emotional characteristics with the target stimuli. Both older and young adults responded slower to targets when distractor valence was incongruent with target valence relative to when distractors were neutral. Older adults responded faster to positive than to negative targets but did not show increased interference effects from positive distractors. In Experiment 2, affective distractors were task irrelevant as the target was a three-digit array and did not share emotional characteristics with the distractors. Twenty-six older (63–84 years) and 30 young adults (18–30 years) gave speeded responses on a digit disparity task while ignoring the affective distractors positioned in the periphery. Task performance in either age group was not influenced by the task-irrelevant affective images. In keeping with the socioemotional selectivity theory, these findings suggest that older adults preferentially process task-relevant positive non-face images but only when presented within the main focus of attention.

## Introduction

A substantial body of empirical research demonstrates that healthy aging is accompanied by declines in cognitive functioning in multiple domains (Gazzaley and D’Esposito, 2007; Gazzaley et al., 2008; Ballesteros et al., 2009). Age-related impairment has been demonstrated in tasks measuring processing speed, working memory, long-term memory, attention, reasoning, and problem solving (Drag and Bieliauskas, 2010; Goeleven et al., 2010; McAvinue et al., 2012). Despite the fact that many cognitive functions do decline with age, others remain relatively stable and a few even improve over the individual’s life course (Ballesteros et al., 2009; McAvinue et al., 2012). A particularly interesting finding in the cognitive aging literature is that emotional control and stability appear to improve with age, with older adults experiencing reduced negative affect, and stable or increased positive affect (Mroczek and Kolarz, 1998; Goeleven et al., 2010; Suri and Gross, 2012; Nicol et al., 2013). One theory that has been proposed to account for the apparent increase in positivity of older adults is the socioemotional selectivity theory (Carstensen et al., 1999). This theory suggests that, as we age, we become increasingly aware that our lifespan is finite and this leads to an increasing prioritization of social and emotional goals (Carstensen et al., 1999; Mather and Carstensen, 2005).

One way such a positive emotion regulation strategy might be realized is by giving attentional preference to positive information, and avoiding or suppressing negative information (Carstensen et al., 1999; Kisley et al., 2007). In keeping with this notion, studies of visual attention have suggested young and older adults may differ in selective attention for emotional information. For example, Mather and Carstensen (2003) tested young and older adults in a dot-probe task in which participants received a simultaneous presentation of two images of faces, one expressing a happy or negative expression (e.g., anger or sadness) and the other, a neutral expression. Older adults responded faster to a dot previously covered by a happy face when paired with a neutral face, and to a neutral face paired with an angry or sad face (Mather and Carstensen, 2003). Young adults, in contrast, showed no attentional preference for any emotional expression. Older but not young adults also show a gaze preference toward happy and away from sad or angry faces when eye movements are tracked (Isaacowitz et al., 2006a,b). These findings are consistent with the argument that goal-directed, controlled processing underpins the positivity effect seen in older adults (Mather and Carstensen, 2005).

The research findings described above suggest the positivity effect is the result of regulation strategies that occur at late stages of processing and require full cognitive control. In line with this premise, the socioemotional selectivity theory maintains that automatic emotion detection processes remain relatively constant over the lifespan of the adult and are largely inaccessible to cognitive control (Mather and Carstensen, 2005). This suggests that the automatic detection of negative information seen in young adults (e.g., Hahn et al., 2006) would be preserved in older adults. Findings from visual search tasks offer mixed evidence. When searching through an array of faces expressing emotions in search of a discrepant face, older adults do not orient toward happy target faces; both older and young adults show an advantage for detecting an angry expression rather than a happy or neutral expression (Hahn et al., 2006). Relatedly, this age-independent ‘anger superiority effect’ was also found in search tasks involving angry, sad or happy faces (Mather and Knight, 2006; Ruffman et al., 2009). Older adults also do not show a positive valence bias in detection of positive, neutral, and negative images in a visual search task, with an overall detection advantage found for emotional over neutral images (Leclerc and Kensinger, 2008). However, Hahn et al. (2006) also observed that older adults differed from young adults in that they were faster to search through arrays of angry faces for a non-angry face compared to searches through arrays displaying predominately happy or neutral faces. In contrast, young adults were slowest when searching through predominately angry face arrays. This suggests that whereas the automatic detection of negative information may be relatively insensitive to aging, the ability to disengage from negative information may be facilitated as part of the aging process (see also Rösler et al., 2005). Consistent with this view are findings from emotional Stroop tasks in which reaction time to name the font color of negative emotional words is typically slowed compared to neutral words (Phaf and Kan, 2007). Older and young adults showed comparable levels of interference from negative emotional words but older adults did not show the carry-over effects of slowing from negative words onto neutral words in consecutive trials that was found with young adults (Ashley and Swick, 2009).

Other methodology (e.g., divided attention tasks) has been used to address the question of whether the strategic processes giving rise to the positivity effect require full cognitive control. Because controlled processes are resource-demanding they can be impaired by competing processes that also place demands on limited attentional resources (Carretié, 2014). Accordingly, any positivity effects seen when full attentional resources are available should be decreased or eliminated in divided attention tasks when attentional resources are limited. Consistent with this premise, Knight et al. (2007) found that the positivity effect demonstrated by older adults under a full attention condition was reversed when the older adults simultaneously performed an unrelated secondary task. In contrast, using a within-subjects design, Allard and Isaacowitz (2008) found that older adults showed a fixation preference for positive and neutral non-face images in comparison to negative images regardless of whether the images were viewed alone or in competition with an auditory task. Relatedly, Thomas and Hasher (2006) found that when young and older adults viewed incidental emotion words while performing a digit parity task, older adults later remembered more of the positive than negative words. Taken together, these results, while mixed (see also, Murphy and Isaacowitz, 2008), suggest that positivity effects may not always necessitate full, cognitive control (Allard and Isaacowitz, 2008).

Recent work suggests that older adults’ preferential processing of positive emotional information may extend to stimuli presented outside the main focus of attention. Ebner and Johnson (2010) looked at the extent to which to-be-ignored emotional faces interfered with young and older adults’ performance of a cognitive task unrelated to faces. Participants were presented with three-digit arrays on a single background face expressing happiness, anger or no expression (neutral) and were instructed to identify the position of the digit that differed from the other two in the array. Trial difficulty was manipulated to include easy and difficult trials. Ebner and Johnson’s (2010) general findings were that the task-irrelevant faces interfered with both young and older adults’ performance on the digit task, that the level of interference varied as a function of facial expression and age of face, and that young and older adults showed different patterns of interference from emotional faces. Specifically, older adults had longer response times (RTs) when happy compared to neutral or angry faces appeared in the background, but only on easy trials. RTs did not differ for angry or neutral faces. Young adults’ responses in the same experiment were slowed down only by angry young faces in comparison to angry older faces on difficult trials. Older adults’ increased interference from happy faces compared to neutral faces suggests that older adults are less able to ignore or disengage from positive stimuli. Moreover, the findings suggest that age-related changes in responses to emotional stimuli may play a role in preferential processing of not only attended but also to-be-ignored positive information.

Using emotional faces as stimuli in a modified version of the negative priming paradigm, Goeleven et al. (2010) also observed age-related differences in processing affective information presented outside the focus of attention. They found that compared to young adults, older adults exhibited lower levels of interference from sad faces. Similarly, inhibition of sad faces was reduced in older adults. In contrast, young and older adults did not differ in the level of interference from or inhibition of happy faces. Goeleven et al. (2010) concluded that increased suppression of negative stimuli may contribute significantly to explanations of age differences in emotional regulation. Greater inhibition could suggest an age-related shift in allocation of attentional resources because older adults exhibit lower levels of active suppression generally in selective attention (Gazzaley and D’Esposito, 2007; Sawaki et al., 2012).

The combined results of Ebner and Johnson (2010) and Goeleven et al. (2010) reveal age-related differences in processing of to-be-ignored, affective information that suggest older adults show an attentional preference toward positive information and/or improved disengagement from or suppression of negative information. As such, they add weight to the argument that the positivity effect may not be wholly reliant on controlled attentional processes. A particularly interesting question related to these findings is whether the effects observed with to-be-ignored stimuli are unique to emotional faces as a class, or whether they generalize to non-face affective stimuli. Numerous studies suggest that faces, as emotionally significant stimuli, preferentially capture or hold attention in comparison with many other classes of stimuli (for a review, see Palermo and Rhodes, 2007). There is also evidence that face and non-face affective stimuli may be processed differently by young and older adults. Ruffman et al. (2006), for example, found that when young and older adults rated images of faces and situations in respect to the level of ‘dangerousness’ they represented, the two age groups differed in respect to how they differentiated between high and low threat faces but not high and low threat situations. Additionally, older adults have significant difficulty identifying negative facial expressions of fear, anger and sadness but readily identify happy facial expressions (see Ruffman et al., 2008, for a review). Older adults’ decreased ability to correctly categorize negative facial expressions could also have played a role in the reduced interference from angry and sad faces observed in the Ebner and Johnson (2010) and Goeleven et al. (2010) studies, further raising the possibility that the results of the two studies may not extend to non-face affective stimuli (see also Wurm et al., 2004). Thus, the primary focus of the current research was an investigation of young and older adults’ processing of distracting, non-face emotional images presented outside the main focus of attention.

A second consideration in the present research relates to task relevance. A number of studies have claimed that processing of to-be-ignored, non-face information is contingent on the relevance of the distracting information to the target and the task (Gronau et al., 2003; Lichtenstein-Vidne et al., 2007, 2012). Lichtenstein-Vidne et al. (2012) asked young participants to indicate the spatial location of a target image that was presented above or below fixation while ignoring emotional, non-face images presented in the periphery. The location of to-be-ignored distractors was either congruent or incongruent with the location of the target, and as expected, distractor location impacted on responses, with slower responses in the incongruent condition. Of particular interest was the effect of the *emotional valence* of the distractors on the target location task. When the target image was neutral, the valence (neutral, positive, or negative) of the distractor images did not impact target location responses. Only when the target was a positive or negative image did the distractor valence interfere with task performance. This led Lichtenstein-Vidne et al. (2012) to conclude that emotional stimuli do not capture attention unconditionally when presented outside the main focus of attention, with processing of to-be-ignored stimuli dependent on their relevance to the task. Specifically, when a to-be-ignored emotional distractor and a target share emotionality as a stimulus feature (e.g., both are emotional images), emotionality becomes task-relevant and distractor valence influences performance of the focused attention task, even when the task does not require processing of emotion (Lichtenstein-Vidne et al., 2012). In contrast, when an emotional distractor and the target do not share this feature (e.g., emotional distractor, non-emotional target), then the influence of distractor valence is null. To our knowledge, there has been no previous investigation of the influence of task relevance on older adults’ processing of distracting emotional information presented outside the focus of attention. A number of studies suggest that there is a general deterioration in attentional control with age (e.g., Roux and Ceccaldi, 2001; Troyer et al., 2006; Anguera et al., 2013), particularly with respect to the active suppression of task-irrelevant information (Machado et al., 2009). This raises the possibility that older adults may be less able than young adults to ignore or disengage from to-be-ignored stimuli, even when task requirements and target characteristics render the distractors task irrelevant.

The central aim of the current study was to investigate age-related differences in the processing of non-face affective images presented outside the main focus of attention. Our primary goal was to address the question of whether or not older adults differentially process to-be-ignored positive and negative valenced images, with a bias toward positive images. In using non-face affective stimuli, we provided an important extension to previous work investigating faces, given that emotional information is communicated not only through facial expressions but also through rich and complex scenes encountered in everyday life. We also investigated the role of task relevance in processing distracting emotional stimuli and, in doing so, further contributed to our understanding of possible changes in visual selective attention associated with healthy aging. Across two experiments we sought to determine the effect of participant age on interference from to-be-ignored affective stimuli using emotional images taken from the International Affective Picture System database (IAPS; Lang et al., 2008). Participants made speeded responses to target stimuli flanked by to-be-ignored IAPS images. The distractors were positive, negative, or neutral images and their task relevance was varied across the two experiments. Hypotheses regarding young and older adults’ performance across the task-relevant and task-irrelevant experimental tasks are detailed in the introductions to the two experiments.

## Experiment 1

Experiment 1 investigated whether task-relevant emotional information presented outside the central focus of attention differentially impacts on young and older adults’ performance on a valence judgment task. Participants made negative or positive valence judgments about centrally located target images taken from IAPS, and the target images were flanked above and below by negative, neutral, or positive images that were to be ignored. The distractor images were task relevant in the sense that they shared similar characteristics to target stimuli (i.e., both were non-face images that contained affective information) and emotional valence was relevant to the requirements of the task.

Three levels of congruency were achieved through all combinations of target and distractor valence: Congruent (negative target and negative distractors, or positive target and positive distractors); neutral (target valence was either positive or negative, and the distractor image was neutral); or incongruent (negative target and positive flankers, or positive target and negative distractors). This experimental design allowed us to examine both facilitation and interference effects and to test the view that any positivity preference in older adults may not be wholly reliant on controlled attentional processes. Facilitation effects are evidenced by faster responding on congruent compared to neutral trials, and interference effects are revealed in slower responses on incongruent compared to neutral trials. On the basis of previous findings, we expected that young adults’ performance on the target valence task would be influenced by the valence of the task-relevant, to-be-ignored flankers, with possibly more impact from negative than positive images (Lichtenstein-Vidne et al., 2012). With regard to older adults, previous findings have suggested that they show an attentional preference toward positive faces and/or improved disengagement from or suppression of negative faces when emotional faces are presented as distracting stimuli (Ebner and Johnson, 2010; Goeleven et al., 2010). If this older adult positivity effect extends to non-face emotional stimuli located outside of the main focus of attention, then we would expect to find greater facilitation and/or interference from positive than negative distractors for older participants.

### Method

The studies were carried out in accordance with APA guidelines and were approved by the University of Otago Human Ethics Committee (reference code D15/159). All participants gave written informed consent in accordance with the Declaration of Helsinki prior to experimental participation.

#### Participants

Thirty older adults and thirty young adults participated in the experiment. Older adults were recruited from databases of older adults research participants maintained by the Face Research Laboratory, University of Otago. Young adults were recruited through the University of Otago, Psychology Department’s experimental participation website and were high school graduates undertaking undergraduate study. Older adult participants were offered $20 and young adult participants $15 as compensation for travel costs. All participants had normal or corrected-to-normal vision, and none had any history of neurological insult. Older adults were screened for cognitive impairment using Addenbrooke’s Cognitive Examination-Revised (ACE-R; Mioshi et al., 2006). The depression subscale of Depression Anxiety Stress Scales – 21 (DASS-21; Lovibond and Lovibond, 1995) was used to assess emotional state. After exclusions based on predetermined criteria (see Results), the final experimental sample consisted of 27 older adults (18 women; age range 64–90 years, *M* = 73.7, *SD* = 6.6), and 25 young adults (15 women; age range 19–29 years, *M* = 22.7, *SD* = 3.3). Educational attainment for older adults was categorized as: primary school (*n* = 1), some high school (*n* = 4), high school certificate (*n* = 4), trade certificate (*n* = 1), technical certificate (*n* = 3), undergraduate degree (*n* = 7), graduate degree (*n* = 5), and education level not provided (*n* = 2).

#### Stimuli and Materials

The emotional images used as targets and flankers for stimulus displays were taken from the IAPS standardized affective images (Lang et al., 2008). IAPS is a set of more than 1,000 images with standardized ratings of valence, arousal, and dominance. Images with large variation in valence ratings (*SD* > 1.6 for positive and negative images; *SD* ≥ 1.2 for neutral images), or images deemed to be culturally specific (e.g., an American style fire hydrant) were excluded from selection. Ninety negative images, 90 positive images, and 45 neutral images were selected on the basis of valence ratings. The overall mean valence rating for the negative, neutral, and positive image sets was 2.0, 5.0, and 7.6, respectively (IAPS numbers for the three categories are given in Appendix). Neutral images were carefully selected to ensure they contained no positive or negative information. The corresponding mean arousal ratings for the three image sets was 6.1, 2.9, and 4.8. The 90 images from each negative and positive valence category were further divided into two subsets of 45 images (i.e., positive subsets a and b, and negative subsets a and b), with subsets a and b equated for mean valence. All images subtended 4.0° × 4.0° of visual angle.

An Intel-PC computer with a 17″ Viewsonic Professional series PT795 CRT monitor with a resolution of 1024 × 768 pixels and a refresh rate of 85 Hz, was used to present stimuli. The presentation of stimulus displays was controlled using E-Prime software (Schneider et al., 2002) and responses were made using an E-Prime response box.

#### Procedure

**FIGURE 1:**
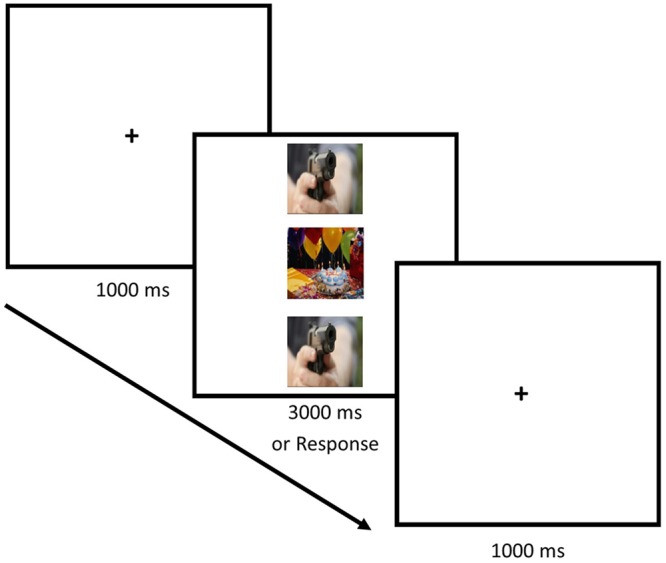
**A typical trial in Experiment 1.** This example depicts a positive valence target flanked by negative valence distractors (incongruent condition). Participants were instructed to indicate the valence of the target image and ignore the distractors. The images are representative and not taken from the IAPS.

Each trial in the experiment began with the presentation of a fixation cross located in the center of the screen and displayed for 1000 ms. The fixation cross was replaced by a stimulus display comprising a target image centered on the fixation cross, and two identical flanker images presented directly above and below the target image. Flankers were presented with their center 5.1° above and below the target center. Participants were instructed to attend only to the centrally presented target image and to ignore the flanker images. On each trial participants were required to indicate as quickly and accurately as possible whether the target image had negative or positive valence by pressing keys on the response box labeled ‘N’ or ‘P’ with the index finger of the left and right hands, respectively. The target and flanker display remained on the screen for 3000 ms, or until the participants responded by pressing a valid response key. **Figure 1** depicts an example trial from Experiment 1.

Six trial conditions were created by the combination of two levels of target valence (negative, positive), and three levels of trial congruency (congruent, incongruent, neutral). The target image displayed in the center of the screen was either positive or negative, and flanked by images that were either congruent (i.e., the valence of the target and flankers matched), or incongruent (i.e., the valence of the target and flankers did not match), or neutral (flanker was neutral). In each of two blocks of 270 trials there were equal numbers of congruent, neutral and incongruent trials, and positive and negative targets occurred equally often within each congruency condition in each block. The assignment of the 45-image subsets to target or flanker categories was counterbalanced across trial blocks. The same neutral images acted as flankers in both trial blocks. Each of the target images appeared three times within a block, once for each trial congruency condition, and each flanker image appeared twice, paired once with a positive target, and once with a negative target. Trials were randomly presented with the constraint that there was never more than two consecutive trials of the same trial condition. Trial blocks were separated by a short self-timed break.

Prior to the experimental trials, visual acuity was tested. All participants completed six practice trials using images not used in the experimental trials to ensure they were familiar with the experimental task and its requirements. Following the experimental trials participants were asked to complete the DASS-21, and older adults additionally completed the ACE-R.

### Results and Discussion

One young participant had a DASS-21 depression subscale score above the cut-off score of 11. After exclusion of this participant, the mean DASS-21 depression subscale scores were 2.0 for older adults and 3.6 for young adults. All older adults scored above the normative cut-off score of 82 on the ACE-R, resulting in a mean ACE-R score of 93.6. Accuracy in experimental trials was calculated for older adult and young adult participants, and data relating to three older and four young participant who failed to meet the 80% accuracy criterion were excluded from further analysis.

Response times < 300 ms were considered anticipatory and excluded from analysis. This resulted in a loss of less than 1% of the data. Mean correct RTs were analyzed using a 2 (age group: older adult, young adult) × 2 (target valence: negative, positive) × 3 (congruency: congruent, incongruent, neutral) mixed-model analysis of variance (ANOVA), in which the between-subjects factor was age group, and the within-subject factors were target valence and congruency. As can be seen in **Table 1**, there was a main effect of congruency *F*(2,100) = 3.20, ρ = 0.05, $\eta_{p}^{2}$ = 0.06. Bonferroni planned comparisons revealed that responses in the incongruent condition (*M* = 848 ms) were significantly slower relative to the neutral condition (*M* = 838 ms), *p* < 0.05. Incongruent responses were also slower compared to congruent responses (*M* = 840), but this difference was not reliable, *p* < 0.10. There was no significant RT difference between the congruent and neutral conditions.

**Table 1:** Mean reaction times in ms (SE) for young and older participants as a function of target valence and trial congruency in Experiment 1.

		Trial congruency		
Age group	Target valence	Congruent	Neutral	Incongruent
Young adults	Negative	728 (26)	726 (25)	745 (27)
	Positive	746 (31)	747 (31)	751 (32)
Older adults	Negative	987 (25)	989 (24)	998 (26)
	Positive	896 (30)	889 (30)	895 (31)

There were also main effects of age group, *F*(1,50) = 30.52, *p* < 0.001, $\eta_{p}^{2}$ = 0.38, and target valence, *F*(1,50) = 7.99, ρ < 0.01, $\eta_{p}^{2}$ = 0.14, and a significant interaction between the two factors, *F*(1,50) = 14.86, ρ < 0.001, $\eta_{p}^{2}$ = 0.23. Planned paired *t*-tests indicated that older adults responded faster to targets with positive valence (*M* = 894 ms) than with negative valence (*M* = 992 ms), *t*(26) = 4.45, ρ < 0.05. In contrast, there was no significant difference in young adults’ responses to positive (*M* = 748 ms) and negative targets (*M* = 733 ms), *t* < 1. No other interaction effects were significant, all *F* < 1.

Mean proportion correct was examined in an identical ANOVA to that conducted for RT. There was no indication of any speed-accuracy trade-off. A main effect of age group revealed that young adults (*M* = 0.97) were more accurate than older adults (*M* = 0.93) overall, *F*(1,50) = 24.78, *p* < 0.001, $\eta_{p}^{2}$ = 0.33. Age group also interacted significantly with congruency, *F*(1,50) = 5.25, ρ < 0.01, $\eta_{p}^{2}$ = 0.10. Bonferroni planned comparisons revealed that the interaction resulted from significantly worse accuracy in the incongruent (*M* = 0.933) than in the congruent condition (*M* = 0.936) for older adults, *p* < 0.05. There were no other significant effects.

In sum, there are a number of notable findings. First, the emotional valence of task-relevant, to-be-ignored flankers influenced task performance. This is line with our expectation that when target and distractors share characteristics such as emotional valence (i.e., task-relevant conditions), distractors presented in the periphery will capture attention and impact target processing (Lichtenstein-Vidne et al., 2012). In addition to target and distractors sharing emotional characteristics, task relevance was enhanced in the present study by requiring judgments of target valence. In this context, positive and negative valence information from the distractor stimuli directly competed with valence information provided by the target on incongruent trials. As expected, this resulted in interference effects that were shown by both older and young adults who responded slower to targets when distractor valence was incongruent with target valence relative to when distractors were neutral. Our expectation that older adults would show greater disruption from positive than negative distractors was not met. This stands in contrast to the finding that older adults responded faster to positive than negative *target* images. Taken together, these latter two results are in keeping with the view that that older adults give preference to positive over negative information only under conditions of full cognitive control, and preferential processing of positive emotional information does not extend to stimuli presented outside the main focus of attention (Carstensen et al., 1999).

## Experiment 2

In Experiment 1, the distractors were task relevant, as both target and distractors were affective images, and the task required a valence judgment. In Experiment 2 there was no task relevance associated with target and distractor characteristics or task requirements, and we examined (1) whether non-face affective distractors affect older adults’ performance of an unrelated directed attention task and (2) whether any processing of such information reflects an age-related positivity bias. We tested young and older adults on a version of the task used by Ebner and Johnson (2010), with three-digit target displays flanked by positive, negative, or neutral IAPS images. Ebner and Johnson (2010) found that older adults showed task-irrelevant interference effects from positive distracting faces but only when task demands were relatively light. Changes in attentional load derived from task difficulty have been shown to influence task performance (Lavie, 1995), and may impact differently on older compared to young adults. Accordingly, we followed Ebner and Johnson and varied the overall task difficulty of the task to include easy and difficult trials. Based on the findings of Lichtenstein-Vidne et al. (2012), we expected to find that for young adults, the task-irrelevant affective flankers would not distract from the digit task in either level of difficulty. For older adults, we hypothesized that if older adults are less able to ignore or disengage from task-irrelevant distractors (e.g., Machado et al., 2009) and the positivity effect does not necessitate full cognitive control (Isaacowitz et al., 2006b; Ebner and Johnson, 2010), then the distractors would impact performance, and positive distractors would disrupt older adults’ performance more than negative distractors on low-task difficulty trials (cf. Ebner and Johnson, 2010).

### Method

#### Participants

Thirty older and thirty young adults were recruited as described in Experiment 1. After exclusions based on predetermined criteria (see Results), the final experimental sample consisted of 26 older adults (16 women; age range 63–84 years, *M* = 72.9, *SD* = 5.6), and 30 young adults (18 women; age range 18–30 years, *M* = 22, *SD* = 2.6). Educational attainment for older adults was categorized as: some high school (*n* = 2), high school certificate (*n* = 4), trade certificate (*n* = 1) technical certificate (*n* = 1), undergraduate degree (*n* = 9), graduate degree (*n* = 7), and education level not provided (*n* = 2). The young adults were all high school graduates undertaking undergraduate study. All participants had normal or corrected-to-normal vision and had not suffered any neurological insult.

#### Stimuli and Materials

Ninety emotional IAPS images subtending 5.7° × 5.7° of visual angle were used as flanker stimuli to create negative, neutral, and positive image sets. The resulting 30-image sets had mean valence ratings of 2.0, 5.0, and 8.0, for negative, neutral, and positive conditions, respectively, with corresponding mean arousal ratings of 6.1, 2.9, and 4.9. An additional set of 10 neutral images not used in the experimental neutral set was created for use as flankers in practice trials. The digits ‘0’, ‘1’, ‘2’, and ‘3’ were used as target stimuli and were presented in Arial font. Large digits were presented in 16 pt. font, and small digits were presented in 10 pt. font.

#### Procedure

Each trial in the experiment began with the 1000 ms presentation of a fixation cross located in the center of the computer screen. The cross was replaced by a stimulus display comprising a target array of three digits presented horizontally and centered on the fixation cross, and two identical flanker images presented directly above and below the target image. Flankers were presented with their center 5.3° above and below the target center. Participants were instructed to attend only to the digit array presented in the center of the screen and to ignore the flanker images. The three-digit array was composed of two distracter digits that matched each other on face value and one target digit that differed from the distracter digits in regard to face value. For each trial, participants were required to indicate as quickly and accurately as possible which of the three digits presented did not match the other two in respect to face value. Participants were required to respond by pressing a key labeled with the corresponding digit (‘1’, ‘2’, or ‘3’) on an E-Prime response box with the first three fingers of their dominant hand. The array and flanker images remained on the screen for 3000 ms or until the participant responded.

**FIGURE 2:**
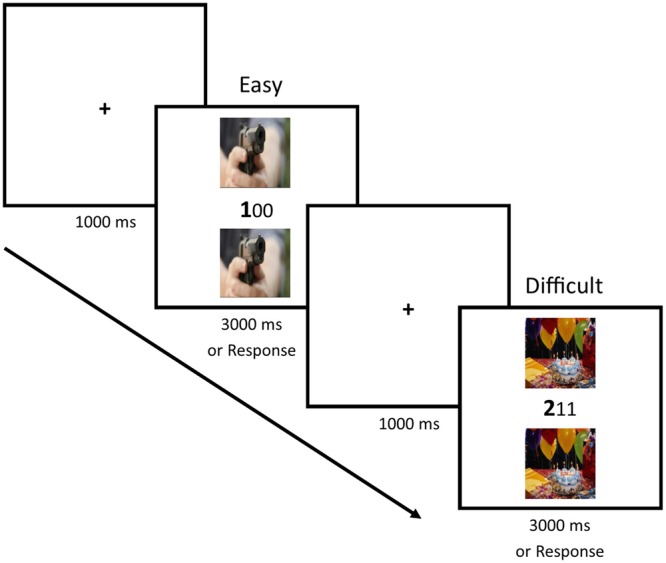
**A typical trial series in Experiment 2.** Participants were instructed to indicate the mismatching digit and ignore the distractors. The example shows two levels of task difficulty, and the correct responses are 1 (Easy trial) and 2 (Difficult trial).

Six trial conditions were created by the combination of three levels of flanker valence (negative, neutral, and positive), and two levels of task difficulty (low and high). In the low difficulty condition, distracter digits were always 0s, distracters were always presented in a smaller font than the target digit, and the target digit’s face value always matched its position within the array (i.e., 100, 020, 003). In the high difficulty condition, distracter digits were randomly assigned (but never matched the target digit) and were 1s, 2s, or 3s. The target digit’s face value never matched its relative position within the array, and the font size (large or small) for each digit within the array was randomly assigned (e.g., 212, 332, 311). **Figure 2** depicts an example trial from Experiment 2. Participants were informed that the experiment would involve an intermixed series of both low and high difficulty trials, and that the procedure and task requirements for each did not differ.

Flanker stimuli were randomly selected for each trial from either the positive, neutral, or negative valence sets of IAPS images. Each image was used twice in each of two blocks of 180 trials, once in a low and once in a high difficulty trial. Within each block, each of the six trial conditions was presented in quasi-random order so there was an equal number of each trial condition and there was never more than two consecutive trials of the same condition.

All participants completed 10 practice trials using neutral images that were randomly selected from a set of images not used in the experimental trials. Practice trials were repeated if the participant did not meet the predetermined accuracy criterion of 80% correct.

### Results and Discussion

One older participant had a DASS-21 depression subscale score above the cut-off score of 11 and two additional older adults had ACE-R scores that fell below the normative cut-off score of 82. After exclusion of these participants, the mean DASS-21 depression subscale score was 1.7 and 2.7 for older and young adults, respectively, and the mean ACE-R score for older adult participants was 93.4. Accuracy in experimental trials was calculated for older and young adult participants, and the data relating to one final older participant who failed to meet the 80% accuracy criterion were excluded from further analysis.

Response times < 300 ms were considered anticipatory and excluded from analysis. This resulted in a loss of less than 1% of the data. Mean correct RTs are shown in **Table 2**. RTs were examined in a 2 (age group: older adult, young adult) × 2 (trial difficulty: easy, hard) × 3 (flanker valence: negative, neutral, positive) mixed-model ANOVA in which the between-subjects variable was age and the within-subject variables were trial difficulty and flanker valence.

**Table 2:** Mean reaction times in ms (SE) for young and older participants as a function of trial difficulty and flanker valence in Experiment 2.

		Flanker valence		
Trial difficulty	Age group	Negative	Neutral	Positive
Easy	Young adults	583 (24)	575 (25)	578 (27)
	Older adults	939 (26)	944 (27)	942 (29)
Hard	Young adults	883 (29)	890 (31)	878 (34)
	Older adults	1326 (31)	1343 (33)	1329 (36)

The analysis revealed an significant main effect of trial difficulty, *F*(1,54) = 905.40, ρ < 0.001, $\eta_{p}^{2}$ = 0.95. As expected, participants responded slower on hard compared to easy trials. The main effect of age group was significant, with older adults responding more slowly than young adults, *F*(1,54) = 104.88, ρ < 0.001, $\eta_{p}^{2}$ = 0.66. The trial difficulty and age group factors also interacted significantly, *F*(1,54) = 13.88, ρ < 0.001, $\eta_{p}^{2}$ = 0.20. Paired comparisons indicated that the significant increase in mean RT for hard trials compared to easy trials was greater for older adults (*M* = 391 ms) than for young adults (*M* = 305 ms), *t*(54) = 3.72, ρ < 0.001. The interaction of flanker and trial difficulty did not reach significance, *F*(2,108) = 2.34, ρ = 0.10, $\eta_{p}^{2}$ = 0.04, and this was the case for all remaining interactions, *F* < 1.

Mean proportion correct was examined in an identical ANOVA to that conducted for RT. No evidence of any speed-accuracy trade-off emerged. Accuracy was high for both older (*M* = 0.98) and young adults (*M* = 0.97) and did not differ across age group, *F*(1,54) = 2.91, ρ = 0.09, $\eta_{p}^{2}$ = 0.05. Performance was more accurate on easy (*M* = 0.99) than on hard trials (*M* = 0.96), *F*(1,54) = 79.19, ρ < 0.001, $\eta_{p}^{2}$ = 0.60. There were no other significant effects (all *F* < 1).

In sum, under task-irrelevant conditions, performance on a digit identification task was not influenced by to-be-ignored affective images presented outside the focus of attention, regardless of participant age or task difficulty. The results with young adults are consistent with previous findings showing that when target and distractors do not share emotion characteristics, distractors fail to capture attention and interfere with target processing (Lichtenstein-Vidne et al., 2012). Importantly, we observed that older adults appear no more vulnerable to the emotional valence of the distractor images than young adults when emotional valence is task irrelevant, even under low-difficulty conditions. Finally, in keeping with the socioemotional theory (Carstensen et al., 1999), older adults did not show a preference toward positively valenced stimuli when the task-irrelevant, emotional stimuli were presented in the periphery.

## General Discussion

In two experiments, we examined whether there are age-related changes in processing non-face affective stimuli presented outside the main focus of attention. Specifically, we addressed the question of whether or not older adults differentially process to-be-ignored positive and negative valenced images, with a bias toward positive images. We also considered whether or not older adults differ from young adults in responding to emotional distractor information as a function of the relevance of the distractor to the target. Our work revealed three relevant findings. First, we found that older and young adults’ performance on a valence categorization task was disrupted by task-relevant emotional distractors. Neither age group showed any evidence of preferential processing of positive (or negative) distractors. Second, we found that older adults responded faster to positive than to negative targets, a positivity response preference that was not present in young adults. Third, we found that neither older nor young adults were distracted by task-irrelevant emotional images when performing an unrelated digit task.

According to the socioemotional selectivity theory, older adults preferentially attend to positive information as part of an adaptive strategy that allows positive affective experience to be maximized (Carstensen et al., 1999). In Experiment 1, when older adults engaged controlled processes to categorize the valence of an image that was the focus of attention, a clear response-time advantage for positive *target* images was found. The processing bias shown by older adults is consistent with the view that with age comes increased motivation to regulate emotion and prioritize processing of positive over negative information (e.g., Carstensen and Mikels, 2005; Mather and Carstensen, 2005). This observed preference for positive over negative attended information was not accompanied by any results to suggest a similar attentional preference toward positive *distractor* images presented outside the main focus of attention. The latter finding is in line with previous work finding no evidence of a older adult positivity effect when attentional resources were limited under divided attention conditions (Thomas and Hasher, 2006; Knight et al., 2007; but see Allard and Isaacowitz, 2008). It is also consistent with previous findings that failed to reveal a positive valence bias in detection of positive, neutral, and negative images in a visual search task (Leclerc and Kensinger, 2008). In conjunction with the observed positive preference for target information, the absence of any observed positivity bias for peripheral distractor information shown by older adults in the present study is consistent with the argument that the strategic processes giving rise to the positivity effect require cognitive control, and that automatic emotion detection processes remain relatively unaffected with advancing age (Mather and Carstensen, 2005).

Our findings seem inconsistent with previous demonstrations that older adults are less distracted by negative than positive distractors when the to-be-ignored background images are faces, and task-relevant (Goeleven et al., 2010) or even task-irrelevant (Ebner and Johnson, 2010). One plausible explanation for these contrasting effects stems from the different class of stimuli used, namely faces in the previous work and non-face images in the present studies. In many cases, faces capture attention or hold attention more readily than non-face stimuli regardless of task relevance (see, Palermo and Rhodes, 2007). For example, face-specific effects in a selective attention task have been reported previously by Machado et al. (2011) who demonstrated that famous faces are subject to less inhibitory processing than non-face stimuli. Because of their particular biological and social relevance (Bruce and Young, 1998), faces may represent a unique category of stimuli that is difficult for both young and older adults to ignore. Facial expression in particular, even when task irrelevant, may be difficult to ignore (Ambron and Foroni, 2015; Ambron et al., 2016). Age-related differences in emotional face processing also could have contributed to the earlier findings of preferential processing of positive distractor information by older adults. Relative to young adults, older adults show performance declines when categorizing negative facial expressions of fear, anger, and sadness but not happiness (Ruffman et al., 2008). They also differ from young adults when judging threat in faces but not scenes (Ruffman et al., 2006). Considered together, these findings suggest that cognitive aging may differentially affect the ability to evaluate emotional stimuli represented by face and non-face stimuli. Our results are consistent with this possibility.

As suggested above, emotional faces may be particularly difficult to ignore even under task-irrelevant conditions, and this may account for the differences in results across studies. However, an additional explanation should be considered. In Ebner and Johnson (2010), the target was centered on the to-be-ignored face, placing distractor information in the center of attention. Previous work has demonstrated that compared to peripheral distractors, fixation distractors are more difficult to ignore and impact more on response selection (Beck and Lavie, 2005) and thus, the positioning of the face distractors at fixation may have advantaged distractor processing in the Ebner and Johnson (2010) study. Lichtenstein-Vidne et al. (2007) have also shown that distractor images presented in the center of attention are hard to ignore regardless of their task-relevance. In a series of experiments using non-affective stimuli they found task-irrelevant, to-be-ignored information affected task performance if it was presented close, but not peripheral to the focus of attention (see also Okon-Singer et al., 2007). When target and distractors are not physically segregated, they may be perceptually grouped and treated as a single entity, with attention allocated to the perceived whole object (Miller, 1991). Thus, differences in the degree of target–distractor segregation could have contributed to the differences found in the current results and in Ebner and Johnson (2010). Future research will be needed to pursue this explanation and determine whether the age-related positivity effect observed with distractor faces presented foveally (Ebner and Johnson, 2010) survives positioning of the distractors outside the main focus of attention. Recent research exploring the impact of task-irrelevant distractors on movement trajectory suggests a promising approach for assessing the positivity effect in older adults in this regard. Ambron and Foroni, 2015 showed that when moving to a target (a dot), young participants’ reaching paths veered toward task-irrelevant distractor faces presented to the left or right of the target but only when the faces expressed anger or happiness relative to neutral expressions. This result demonstrates that emotional faces capture attention even when emotion is irrelevant to the task and the distractors are presented in the periphery. This indirect measure of preferential attention to emotional faces could be effectively used with older adults, employing both face and non-face emotional stimuli, to further probe age-related changes in emotion processing in task-relevant and task-irrelevant contexts.

The absence of any attentional bias in older adults, as suggested by the lack of a positive distractor effect, may not necessarily rule out the possibility that distractor valence was not differentially processed. In an earlier study, Thomas and Hasher (2006) found no evidence of an attentional bias toward positive words when older adults viewed a centrally presented positive, negative or neutral word and made a decision about the parity of two digits that flanked the word; valence did not influence performance in the parity task. Nonetheless, in a subsequent surprise recognition task, older adults recognized positive words but not neutral or negative words at above chance levels. The emergence of this positivity effect in memory in the absence of any early processing biases suggests that post-encoding processes led to the memory effect (Thomas and Hasher, 2006). Further work is needed to clarify the nature of the positivity effect both in memory and attention tasks (Murphy and Isaacowitz, 2008) and it would be informative to test for age-related positivity effects in recognition memory using the current paradigm and class of stimuli in future research.

In keeping with Lichtenstein-Vidne et al. (2012), our results suggest that non-face emotional stimuli do not capture attention unconditionally, with the impact of peripheral distractors on target processing dependent on task relevance. Our study extends the work of Lichtenstein-Vidne et al. (2012) with young adults in finding that older adults’ vulnerability to non-face affective distractors positioned in the periphery may also depend on the relevance of the task. Older and young participants’ performance was adversely affected by distractor images when the target and task requirements made the valence of the distractors relevant. Conversely, when the target and task requirements rendered distractor valence irrelevant the performance of both age groups was unaffected by the irrelevant emotional distractors, regardless of the difficulty level of the primary task (cf. Ebner and Johnson, 2010). Taken together, this suggests that under some conditions, older adults are able to ignore or disengage from task-irrelevant distractors and may be no more vulnerable to distraction than young adults.

Task-relevance was achieved in the present study by having valance feature both as a characteristic shared by the distractors and targets, and as the required judgment in the central task. In accordance with the central aim of the study, this was done to provide optimal conditions for detecting any potential older adult positivity bias in processing of the emotional distractors, as well as the attended target of the central task. It would be of interest in future work to test conditions in which the central task did not require an emotion-based decision and task relevance was achieved solely through the shared emotional characteristics of the distractors and target. This would provide additional insight into possible age-related differences in the degree to which emotional stimuli capture or hold attention when presented outside the attentional focus, and the role that task relevance plays.

More broadly, future investigations could also consider the impact of task-irrelevant emotional expressions on categorization of gender or ethnicity in faces as a means to understanding potential preferential processing of positive emotion information in older adults. Evidence suggests multiple facial features are processed when only one particular feature is explicitly attended to (Mouchetant-Rostaing et al., 2000; Ito and Urland, 2005; Ambron et al., 2016; Li and Tse, 2016). For example, recent work by Li and Tse (2016) with young participants showed that task-irrelevant emotional expressions in target faces interacted with the processing of gender or race when the faces were categorized on the latter dimensions, and Ambron et al., 2016 found that when gender in a distractor face was used to cue the non-face target to be reached for, task-irrelevant facial expression modulated the degree to which reaching paths deviated to the distractor faces. Notably, this effect was particular to facial emotion, as gender did not modulate the reaching trajectory when task irrelevant.

One limitation of the current study is that arousal was lower for neutral images than for positive and negative images, as is typically the case in studies evaluating the effect of valence on behavior. Thus, there is potential that arousal rather than valence explains the observed effects of the distractor stimuli on target processing. This is of particular relevance in Experiment 1 where interference effects were found. Our results suggest this is not the case. An arousal-based interference effect would have required interference effects from emotional distractors that was independent of the valence of the target and distractor stimuli, such that either a positive or negative distractor coupled in any combination with positive or negative emotional targets would produce slower RTs compared to when the distractor was neutral. This was not the case, as the interference effects were found only when target and distractor valence did not match (i.e., positive target, negative distractor and vice versa) and not when target and distractor valence matched (i.e., positive target, positive distractor and negative target and negative distractor). The potential combined role of valance and arousal in older adults’ processing of emotional distractor information could be explored in future work.

Another limitation is that we did not compare face and non-face affective stimuli in our study. Such a comparison would have allowed a direct assessment of the effect of both types of affective stimuli on target processing and the differential effect that facial expressions may have on age-related differences in distractor processing, something that could be addressed in future research.

In summary, the present study investigated differences in young and older adults’ processing of non-face affective images presented outside the main focus of attention. We found that emotional non-face distractor images presented outside the focus of attention interfered with target processing for both young and older adults when the distractor and target shared emotion characteristics. Older adults showed a positivity effect for task-relevant emotional target images, but no evidence for preferential processing of positive distractor images was found regardless of whether distractors were task relevant or task irrelevant. In keeping with the socioemotional selectivity theory (Carstensen et al., 1999), these findings suggest that older adults preferentially process task-relevant positive emotional images but only when presented within attentional focus. Given previous indications that older adults are more distracted by happy than angry, to-be-ignored faces (e.g., Ebner and Johnson, 2010), our results suggest that age-group differences in processing of emotional information outside the main focus of attention may depend upon whether that information is communicated through everyday scenes or facial expressions.

## Author Contributions

MM and JM designed the research, MM collected the data, MM and JM analyzed the data and wrote the manuscript.

## Conflict of Interest Statement

The authors declare that the research was conducted in the absence of any commercial or financial relationships that could be construed as a potential conflict of interest.
