# Supplementary material for: Processing Distracting Non-face Emotional Images: No Evidence of an Age-Related Positivity Effect
Source: Front Psychol. 2017 Apr 13;8:591. doi: 10.3389/fpsyg.2017.00591 (PMC5389978; doi:10.3389/fpsyg.2017.00591)
Supplement: Supplementary file 1 [file Data_Sheet_1.DOCX]

**Appendix**

**Experiment 1**

IAPS numbers of negative valence pictures in set A:

2095, 2205, 2345.1, 3010, 3016, 3030, 3062, 3068, 3100,

3101, 3103, 3110, 3130, 3140, 3150, 3181, 3195, 3230,

3301, 3500, 6021, 6212, 6350, 6520, 6560, 6563, 9075,

9140, 9163, 9183, 9185, 9187, 9252, 9295, 9322, 9340,

9412, 9414, 9420, 9435, 9570, 9902, 9903, 9908, 9921

IAPS numbers of negative valence pictures in set B:

2352.2, 2375.1, 2703, 2799, 2800, 2811, 3000, 3001, 3005.1,

3059, 3060, 3069, 3071, 3120, 3180, 3191, 3225, 3530,

3550.1, 6022, 6230, 6243, 6313, 6415, 6540, 9040, 9220,

9253, 9254, 9301, 9302, 9325, 9332, 9405, 9413, 9421,

9428, 9433, 9571, 9635.1, 9800, 9901, 9904, 9910, 9911

IAPS numbers of positive valence pictures in set A:

1441, 1600, 1630, 1710, 1811, 2035, 2045, 2050, 2057,

2058, 2070, 2080, 2158, 2165, 2170, 2209, 2216, 2222,

2224, 2299, 2304, 2341, 2347, 2388, 2398, 2598, 2660,

4626, 4641, 5001, 5202, 5210, 5600, 5631, 5760, 5825,

5829, 5833, 5910, 8170, 8200, 8210, 8370, 8461, 8470

IAPS numbers of positive valence pictures in set B:

1440, 1460, 1590, 1610, 1620, 1721, 1750, 1920, 1999,

2071, 2091, 2150, 2154, 2260, 2274, 2311, 2314, 2332,

2340, 2530, 2540, 2550, 5200, 5270, 5621, 5623, 5660,

5700, 5779, 5780, 5814, 5830, 5831, 5836, 5891, 7502,

8080, 8185, 8190, 8350, 8380, 8420, 8497, 8499, 8540

IAPS numbers of neutral valence pictures:

1616, 2102, 2393, 2396, 2397, 2411, 2495, 2514, 2516,

2890, 5471, 6150, 7000, 7002, 7004, 7006, 7009, 7010,

7012, 7014, 7018, 7019, 7020, 7034, 7035, 7036, 7037,

7038, 7041, 7045, 7050, 7053, 7055, 7056, 7059, 7160,

7161, 7175, 7179, 7185, 7217, 7235, 7491, 7547, 7705

**Experiment 2**

IAPS numbers of negative valence pictures:

2205, 2352.2, 2703, 2811, 3016, 3030, 3101, 3103, 3180,

3191, 3195, 3230, 6022, 6313, 6350, 6520, 6560, 9163,

9185, 9220, 9252, 9253, 9254, 9325, 9414, 9571, 9635.1,

9800, 9910, 9921

IAPS numbers of positive valence pictures:

1440, 1441, 1460, 1610, 1710, 1750, 1920, 2045, 2050,

2057, 2058, 2070, 2071, 2080, 2150, 2154, 2260, 2340,

2347, 2530, 2550, 5210, 5760, 5825, 5830, 5833, 5910,

8190, 8370, 8420

IAPS numbers of neutral valence pictures:

2102, 2396, 2397, 2411, 2514, 2516, 2890, 6150, 7000,

7002, 7004, 7009, 7010, 7012, 7014, 7019, 7020, 7034,

7035, 7041, 7045, 7050, 7055, 7056, 7059, 7160, 7161,

7179, 7185, 7235

IAPS numbers of neutral valence pictures in practice trials:

1616, 2393, 2495, 5471, 7006, 7036, 7053, 7175, 7491,

7547
